# Examining Gifted Students' Evaluations of Their Education Programs in Terms of Their Project Production and Management

**DOI:** 10.3389/fpsyg.2022.833395

**Published:** 2022-01-31

**Authors:** Gülnur Özbek, Miray Dağyar

**Affiliations:** Department of Educational Sciences, Faculty of Education, Akdeniz University, Antalya, Turkey

**Keywords:** gifted education program, project production, project management, science and art centers, gifted students

## Abstract

The aim of this study is to examine gifted students' evaluations of their education programs in terms of their project production and management by considering the basic principles of gifted education and training programs. In evaluating the effectiveness of programs for gifted students, it is regarded as important to consider the evaluations of the individuals for whom the programs are implemented. Project production and management was taken as the basis for the principles and guidelines of the programs implemented for gifted students. A mixed research design was used in the study. In the quantitative part of the research, the views of 300 randomly selected gifted students, who were attending the project production and management (PPM) and special talent development (STD) programs at Science and Art Centers (SACs) throughout Turkey, regarding their evaluation of the education programs, were analyzed. In the qualitative part of the research, the project production and management of the gifted individuals in the upper and lower groups according to their program evaluations were evaluated descriptively by examining the project proposals they developed. In the analysis of the quantitative data, it was determined that the participants had positive views about the effectiveness of the program, but that there was a significant difference between the upper and lower groups in terms of program evaluation scores. Considering this situation, the data of 10 participants in total (five participants included in each of the upper and lower groups) were subjected to qualitative analysis in the second stage. As a result of the study, it was determined that all of the gifted students in the upper and lower groups were able to produce projects, but that in the categories specified in their projects, there were differences in favor of the upper group in terms of depth, originality, taking their talents into account, acting consciously, acting in accordance with the plan, participating in more prestigious competitions, and quality.

## Introduction

Evaluation of the education program aimed at gifted individuals is important in terms of revealing the effectiveness of the program. The effectiveness of the training program is determined according to the change in the skill (Saygili and Atahan, 2014; Gözeten, 2017; Güneş and Aybek, 2018; Thahir et al., 2019), creativity and productivity (Kim et al., 2016), achievement (Cho et al., 2015; Little et al., 2018), competence (Yu and Yun, 2017), ability (Kök and Davasligil, 2014) and attitude (Deringöl and Davasligil, 2020) observed in the individuals for whom the program is implemented, or according to the original products and projects produced by these individuals (Manuel and Freiman, 2017; Sengil-Akar and Yetkin-Ozdemir, 2020). In addition, taking into account the evaluations of the individuals for whom the programs are implemented and collecting data using a program evaluation scale are regarded as effective in evaluating the effectiveness of programs aimed at gifted students (Sak, 2011; Kayişdag and Melekoglu, 2019).

In studies in the literature on the effectiveness of programs for the gifted, it is seen that the original ideas, products, problem solutions and projects produced by these individuals are examined and it is emphasized that project production can be supported with the programs that are implemented (Callahan et al., 2015; Kim et al., 2016; Manuel and Freiman, 2017; Yu and Yun, 2017; Thahir et al., 2019; Sengil-Akar and Yetkin-Ozdemir, 2020). In addition, when the principles, objectives, relevant legislation and theoretical framework of the education and training programs for the gifted are examined (Betts, 1986; MoNe, 2018, 2019), it can be said that the association of students' program evaluations with their project development overlaps considerably. In the studies in the literature on the determination of the program needs and the evaluation of the programs (Callahan et al., 2015; Kontaş and Yagci, 2016; Özçelik, 2018), the need to determine and analyze the status of the gifted in producing original projects is expressed. In this context, the importance of associating the program evaluations of gifted students with their production of projects emerges.

### Gifted Education Programs and Teaching: The Science and Art Center

Gifted students are individuals who have academic ability, high learning speed, and high-level skills, capacity and performance in one or more areas compared to their peers. Science and Art Centers (SACs) are institutions where gifted individuals receive education in order to develop their talents, skills, competencies and potentials in the fields of science and art, based on their educational needs according to the fields in which they are identified (MoNe, 2019). In the centers, individuals receive training and participate in programs if they are identified in at least one of three different skill areas: general mental, visual arts and music. Advancement in the programs varies according to the field of talent, and it is achieved by the completion of five programs, namely the orientation program in the field of general mental development, the support training program, the individual talent recognition program, the special talent development program, and the project production and management program (MoNe, 2018, 2019). In the orientation program, which is the first stage, students are informed about the physical environment of the SAC, the institution, and the education model implemented in the institution. In the support training program, which is the second stage, the aim is to improve the students' skills in solving problems, conducting scientific research, and using scientific research methods to create projects. In the third stage, the individual talent recognition program, students are allowed to discover their talents by doing activities in different fields. In the fourth stage, the special talent development program, the aim is for students to acquire advanced knowledge, skills and behavior in a discipline by also taking interdisciplinary relations into account. In the project production and management program, which is the last stage, consultancy is provided to students in order to foster knowledge and experience related to the project preparation and development processes.

When the advancement process in the programs is examined, it is seen that by starting from the establishment of interdisciplinary relationships with basic skills, individuals first identify all fields and determine their own talents, then develop their special talents in a field or fields, and as a result, are able to create original projects in line with their interests and wishes in their area of expertise. In this context, it can be stated that advancement in the programs is in a pyramidal structure, which gradually narrows from the general to the specific in line with the individual's abilities, and that it is based on the creation of projects by specializing in one or more fields (MoNe, 2018, 2019). In terms of education programs and instruction, in the project production and management program for gifted students, there are emphases on such areas as developing useful models, obtaining original results, making inferences and interdisciplinary study, individually or with a group (MoNe, 2019).

Differentiation of gifted education programs is defined as shaping education programs according to individual differences in gifted education, and for this purpose, curriculum differentiation models are developed (Akkaş and Tortop, 2015). When different curriculum differentiation models are examined, it is seen that gifted students' creation of a program-based product is taken as the basis (Renzulli, 1977; Maker, 1982; Betts, 1986). In the product component of the Curriculum Differentiation model, Maker (1982) stated that gifted students should create quality products by focusing on their real-life problem solving skills. In the Autonomous Learner model, Betts (1986) emphasized that students should conduct in-depth research, explore, and present the products they create in order to become independent and knowledgeable individuals. In these models, it is seen that the gifted individual is expected to do research and to create and present original products. It can be said that in terms of the fact that one of the ultimate and common principles of the SAC programs is to develop an original product and project, there is similarity with the curriculum differentiation models in this sense. It is emphasized that the planning, implementation and evaluation stages of education and training programs in the SACs in Turkey are carried out in a way that will enable the training of individuals who can produce solutions to real-life problems, conduct scientific research and make inventions (MoNe, 2018, 2019). Considering the principles and guidelines of the education and training programs and the stages of planning, implementation and evaluation (MoNe, 2018, 2019), it can be concluded that there is a strong relationship between the evaluations of the relevant programs and the production and development of projects. For this reason, the relationship, necessity and importance of examining the status of project production and development emerge, as well as the views of gifted students about the effectiveness of and their satisfaction with the program.

When the principles of the project production and management (PPM) program in SACs are examined, it is stated that each individual who attends this program is expected to prepare at least one project for each academic year, that the consultant teacher should prepare at least two progress reports during the project development process, and that information about the completed projects should be processed into the SAC program (MoNe, 2018, 2019). It can be said that examining whether gifted students can produce projects, whether they can develop original products, and also, if the project has been developed, examining these according to certain criteria will provide important data for the effectiveness of the programs as well as the program evaluations of the students.

In the literature evaluating the effectiveness of education and training programs implemented not only for gifted students, but also for other individuals, there are studies examining students' model creation statuses (Tekin Dede and Yilmaz, 2015; Hidayat et al., 2018) and their original solutions to contextual problems that are presented (Hendriana et al., 2019).

This research aims to examine gifted students' evaluations of their education programs in terms of their project production and management by considering the basic principles of gifted education and training programs, and in this respect, it has originality in the literature. This research aims to examine project production according to program evaluation scores. Within the scope of the stated aim, the quantitative research question was determined as: Is there a significant difference between the groups according to the upper and lower group distributions of the views of gifted students regarding their evaluation of the education programs? The qualitative research question was expressed as follows: How is the project production and management of gifted students in the upper and lower groups according to their program evaluations?/How do they differ?/Is there a difference between them?

## Methods

A mixed research design was used in the study (Cresswell and Clark, 2015; Cresswell, 2016). In the quantitative part of the study, the gifted students' views regarding their evaluations of their education programs were analyzed. Survey studies provide the opportunity to generalize by making inferences in terms of certain characteristics (Cresswell, 2016). In the qualitative part of the study, the project production and management of the gifted individuals in the upper and lower groups were evaluated descriptively according to their program evaluations by examining the project proposals they developed.

### Study Groups

#### Quantitative Study Group

The Science and Art Centers (SACs) continue their project production and management (PPM) and special talent development (STD) programs throughout Turkey, and 300 randomly selected gifted students make up the quantitative research group. The Science and Art Centers (SACs) continue their project production and management (PPM) and special talent development (STD) programs throughout Turkey, and 300 randomly selected gifted students make up the quantitative research group. Participants consider as gifted who passed and received high scores through three-stage identification process are selected for education at SACs. In the first stage, students who are gifted are nominated by their class teacher by filling out the “Primary Education-Age Teacher Observation Form.” In the second stage, the Basic Abilities Test is applied to the students nominated. Candidates who show high performance in this test are taken into individual evaluation at the third stage (Dagyar et al., 2022). First study group was selected by simple random sampling and is summarized in Table 1.

**Table 1:** Demographic information of first study group.

**Characteristics**	**Category**	**Participants**	
		***f***	**%**
Gender	Female	115	38.3
	Male	185	61.7
Type of school	Public	218	72.7
	Private	82	27.3
Program level	PPM	261	87
	STD	39	13
Geographical area of residence	Eastern Anatolia	25	8.3
	Central Anatolia	47	15.6
	Black Sea	34	11.3
	Mediterranean	130	43.3
	Aegean	39	13
	Marmara	18	6
	Southeastern Anatolia	7	2.3
Skill area	General mental	300	100
Grade level	6	9	3
	7	12	10.3
	8	4	1.3
	9	96	32
	10	102	34
	11	46	15.3
	12	31	10.3
Age	11	9	3
	12	12	4
	13	10	3.3
	14	90	30
	15	98	32.6
	16	51	17
	17	30	10
Years of experience at SAC	4	34	11.3
	5	28	9.3
	6	31	10.3
	7	93	31
	8	72	24
	9	32	10.7
	10	8	2.7
	11	2	0.7
	Total	300	100.0

When Table 1 is examined, it is seen that there are participants from 7 geographical regions (39 cities and participants attending the centers in these cities are summarized by taking into account the participants' personal information forms). The reasons for the variation in the number of participants according to the regions are the absence or very low number of participants in the last two program levels in SACs, and the inability to enable voluntary participation. Since the principle is progress according to the talent development of the study group, their ages, grade levels, and also years of experience at SACs can differ even if they are at the same program level. Program levels such as PPM are considered and have importance instead of ages and grades of the students at SACs (MoNe, 2018, 2019). Also, it can be stated that participants who are male and who attend public schools constitute the majority.

#### Qualitative Study Group

Outlier sampling which is discussed as a types of purposive sampling techniques was used. It involves selecting participants who are outlier or extreme in terms of scores (Patton, 1987). In the qualitative study group of the research, 10 participants who are outlier or extreme in terms of scale scores were selected from first study group to in-depth research on PPM. Five participants each in lower 27%-upper 27% groups in the first study group were determined purposively in order to examine the PPM processes and the difference between them. The second research question of the study, which aims to examine and evaluate the difference in PPM, was taken into account in the determination of the 10 participants from the lower 27%-upper 27% groups. The second study group is summarized in Table 2.

**Table 2:** Demographic information of second study group.

**Characteristics**	**Category**	**Upper**	**Lower**
		**f**	**f**
Gender	Man	4	3
	Woman	1	2
Type of school	Public	4	2
	Private	1	3
Program level	PPM	5	5
Skill area	General mental	5	5
Geographical area of residence	Mediterranean	5	5
Grade level	9	1	1
	10	2	1
	11	1	2
	12	1	1
Age	13	1	2
	14	1	1
	15	2	1
	16	1	1
Years of experience at SAC	6	1	0
	7	0	1
	8	1	1
	9	1	2
	10	1	1
	11	1	0
	Total	5	5

In addition to the information summarized in Table 2, it was determined that all of the qualitative study group continued in the field of mathematics at the PPM program level.

### Data Collection Tools

#### Quantitative Data Collection Tools

##### Personal Information Form

This includes questions for determining the demographic characteristics of the gifted students: (1) SAC attended; (2) Type of school (public-private); (3) Program level (STD, PPM); (4) Skill area (general mental, music, visual arts); (5) Grade; (6) Geographical area of residence; (7) Age; (8) Years of experience at SAC.

##### Gifted Education Program Evaluations-Student Form

The Gifted Education Program Evaluations-Student Form (GEPE-SF), which was revised and whose psychometric properties were determined, was used (Sak, 2011; Avci, 2015). It is stated that the scale was developed and revised to be used for formative and informative program evaluation for gifted students (Sak, 2011; Avci, 2015). The scale is used in gifted education program evaluations and the psychometric properties of its revised version were evaluated with the data obtained from 319 participants at the 6th, 7th, and 8th grades attending 9 different SACs in 7 different cities. In addition to the education program standards for the gifted, developed by the USA National Association for Gifted Children (National Association of Gifted Children (NAGC), 2010), the differentiations in the program dimensions of the Maker Model, and Van Tassel-Baska's Integrated Curriculum Model were taken into account (Sak, 2011; Avci, 2015). The scale consists of 49 items, of which 4 are negatively worded (6, 8, 41, and 49) and 45 are positively worded. The Cronbach alpha reliability coefficient of the scale, which has a single-factor structure, was calculated as 0.97.

Within the scope of this research, the validity and reliability study of the GEPE-SF was conducted. To obtain results related to the construct validity of the scale, confirmatory factor analysis was performed on the scores obtained from the group to which the scale was administered within the scope of the study. The fit index values and fit levels of the scale (χ^2^ = 3,147.55, df = 1,127, *p* = 0.000, χ^2^/df = 2.79, GFI = 0.96, IFI = 0.96, NFI = 0.94, NNFI = 0.96, CFI = 0.96, AGFI = 0.93, SRMR = 0.05, RMSR = 0.08, and RMSEA = 0.08) were calculated and evidence was obtained that the scale has a valid structure for this sample (Sümer, 2000; Kline, 2005). The Cronbach alpha internal consistency coefficient calculated to determine the reliability of the obtained scores was calculated as 0.94 and was interpreted as reliable for this sample (Özdamar, 2004).

#### Qualitative Data Sources

In order to collect the qualitative data, forms prepared in accordance with the principles and guidelines of the SAC programs and currently in use were used. The items and guiding statements in the forms are aimed at determining project production and management. In addition, within the scope of the research, opinions on the suitability of the forms were received from measurement and language experts. Since the question and guiding statements in the forms were found to be understandable, they were used in their original form.

##### Project Report Form

In this form consisting of a total of 15 items and guiding statements, the aim is to collect data related to the project production process such as the name of the project, results and suggestions. Some of the guiding statements in the form are: “Whether the project has been published anywhere:...” and “Competitions (if any) that the project was entered in and the placings it received:....”

##### Project Production and Management Program Observation and Evaluation Form

This form consists of 3 parts (information about the owner of the project, observation and evaluation items regarding the project, and a brief explanation of the project). There are 21 items in the second part. Some of the items are: “It applies for a patent related to its original product” and “It converts project results into products.”

##### Project Follow-Up Form

This form consists of 3 parts (information about the project owner, project follow-up process, evaluation of the project). In the second part, there are sections about the dates when checks were made in stages from the start date of the project, the stage that was reached in the checks, the result of the check, and whether it was completed or not. Some of the items in the statements in the third part are: “Making a plan according to the project, preparing a timeline: …” and “Carrying out the project according to the plan: ….”

### Data Collection Process

#### Quantitative Data Collection Process

During the data collection, the identity information of the participants was kept confidential, and they were allowed to voluntarily participate in the research and fill in the consent form. The research was carried out in two stages. In the first stage, the quantitative data of the research were obtained by administering the GEPE-SF scale and the personal information form to participants throughout Turkey.

In the second stage, regarding the program in which project production is the basis, data were collected about the project production statuses as well as the program evaluations of gifted students throughout Turkey. The aim of the research was taken into account in making the decision about which participants to collect these data from. In addition to the aim of the research, the points taken into account in the collection of these data were: (1) to determine and examine the project productions of the participants in the upper and lower groups in terms of program evaluations, (2) to determine the province with the highest number of participants in the first stage and gather qualitative data from this participant group. From among the participants, data on the project productions and management of a total of 10 participants, five each in the upper and lower groups in terms of program evaluations, were collected with the program forms of these participants.

#### Qualitative Data Collection Process

Data entry was made during the fall and spring academic semesters to 3 different data sources related to the project production and management processes of the upper and lower group participants according to their program evaluations. Data entries were made in line with progress in the project stages. The fact that project production requires a long time, that there are annual periods for applications for dissemination, and that on the basis of the special talents program, individuals who are in the PPM program are expected to carry out project production during the academic year (MoNe, 2018, 2019) constitute the reasons for the data collection period.

### Data Analysis

#### Quantitative Data Analysis

SPSS 23.0 analysis program were used to analyze the quantitative data obtained within the scope of the research. Before the analysis began, incorrect data entry was checked and the negatively-worded items (6, 8, 41, and 49) in the GEPE-SF scale were reverse coded. Before beginning the analysis, missing values and extreme values were examined. There were no missing values in the data set. As a result of the extreme value analyses, 8 individuals (participant numbers 18, 20, 31, 32, 87, 242, 249, and 250) were removed from the data set and the analyses were continued in this way. Then, the normality of the distributions of the total scores obtained from the scales and the homogeneity of the groups (Levene's Test for equality of variances) were examined for parametric techniques (see **Table 4**). Descriptive statistics were calculated for each score. Considering ranking of the program evaluation scores of the participants, lower 27%-upper 27% groups were determined. *T*-test was employed to determine the differences between the scores of the participants in the lower 27%-upper 27% groups. It is suggested that upper and lower groups consisting of 27% from the extremes of the criterion score distribution are optimal for the study of test items (Kelley, 1939).

#### Qualitative Data Analysis

As shown in Table 3, within the scope of the research, the project production of the participants attending the program was examined by analyzing the qualitative data obtained from the data sources of each participant's (1) Project report form, (2) Project production and management program observation and evaluation form, and (3) Project follow-up form. In the analyses, the data obtained from data sources (2) and (3) were used to confirm and validate the findings obtained from data source (1).

**Table 3:** Sample data analysis and coding.

	**Data analysis and coding**						**Confirmation review**
Data Source	(1) Project report						(2) Project production and management program
Category	Determining the project topic	Determining the project problem situation	Working on the project	Finalizing the project	Disseminating the project	Product	observation and evaluation forms (3) Project follow-up form
Sample coding	-Following national and international competitions	-Preparing a research question - Appropriate for special talent	-Compatible with scientific research methods	-Achieving original mathematical generalizations	-Participating in project-based competitions	-Mathematical generalization	
Sample participant statements	“*The project was inspired by the solution developed to the problem in the international CEMC mathematics contest.”*	“*If the variables and conditions are generalized, how the solutions can be generalized and proven is calculated with combinatorics”*	“*Solutions were modeled with tables and solution generalizations were made via the models. Later, the proof was tested.”*	“*The generalized solution that gives the number of positions was obtained”*	“*It was presented at the TÜBITAK research projects competition for high school students.”*	“*Mathematical generalization of the solution is made together with its proof”*	

The ultimate principle and basis of gifted programs is the production of projects (MoNe, 2018, 2019). In the analysis of the qualitative data, the project production and management of each participant was examined under five categories: (1) Determining the project topic, (2) Determining the project problem situation, (3) Working on the project, (4) Finalizing the project, and (5) Disseminating the project. In addition, the case of whether or not they produced a project/product was determined. In determining these categories, the theoretical framework on which this research is grounded on related with SACs and PPM program principles in terms of project production evaluation was taken into consideration. The principles and guidelines on which the PPM and SACs program are based and program evaluation forms which are qualitative data sources of the research were examined. Existing categories in qualitative data sources such as PPM program observation and evaluation form of the research were used (MoNe, 2018, 2019). Additions were made to the existing categories by taking into account and analyzing the participants' project reports data.

When Table 3 is examined, how the data was analyzed and confirmed, sample participant statements and the coding made for these statements are summarized. The accuracy of the data obtained from the participants' project reports was confirmed in a sense by examining their “project production and management program observation and evaluation forms” and their “project follow-up forms”. For example, the statement “*The geometric proof obtained was presented in the project competition”* in the project report of one of the participants was confirmed by the item “*Participates in project-based competitions*”, the data entry for which was made by the consultant for that participant. Similarly, in the project report, the statement “*It took three months to make trials according to the rules of the move and to generalize the results”* was confirmed by the statement “*Carrying out the project according to the plan”* in the project follow-up form, the data entry for which was made by the consultant for that participant.

The strategies of seeking expert opinion, obtaining participant confirmation, making detailed descriptions, engaging in long-term interaction, and using diversification to increase internal validity (Cresswell and Clark, 2015) were used to ensure the validity of the research. Compatibility between coders and the researcher's own coding, inclusion of direct quotations, and conducting the data analysis based on the principles and guidelines of gifted programs were used to ensure reliability (Miles and Huberman, 1994; Yin, 2009; Yildirim and Simşek, 2016). The arithmetic mean of the Kappa coefficients calculated after independent coding was 0.84 (Landis and Koch, 1977) and the level of inter-coder agreement was 0.94 (Miles and Huberman, 1994), thus determining that the coding was reliable. Inconsistencies were found in the codes for determining the project problem situation and working on the project.

## Findings

### Opinions of Gifted Individuals on the Effectiveness of the Education Program

The analysis results for the participants' program evaluation (PE) scores are summarized in Table 4.

**Table 4:** Independent group *T*-test for the comparison of upper 27% and lower 27% groups.

**Score**	**Group**	**Descriptive**						**Homogeneity**		***T-test***		
		***N***	**%**	***X***	**Min**	**Max**	***S***	***F***	***p***	***t***	**sd**	***p***
PE	Total	292	100	202.57	133	260	24.60	0.078	0.13	32.58	1.83	0.001
	Upper	79	27	230.51	222	260	6.16					
	Lower	79	27	170.65	133	190	15.12					

When Table 4 is examined, it is seen that the participants' mean scores for their program evaluation views can be considered high. These findings can be expressed as that gifted individuals had positive opinions about the effectiveness of the implemented program. In terms of evaluation scores, a significant difference was determined between the upper 27% and lower 27% groups in favor of the upper group (*X* = 230.51).

As stated in the problem situation of the research, the principles and guidelines of the program implemented for gifted students are based on project production and management. In the analysis of the quantitative data, it was determined that the participants had positive views about the effectiveness of the program, but that there was a significant difference between the upper and lower groups in terms of program evaluation scores. Considering this situation, it was decided to examine the findings for project production and management according to the program evaluations of the participants in the upper-lower groups. According to the gifted students' program evaluations, the data of 10 participants in total, five participants each in the upper and lower groups, were subjected to qualitative analysis in the second stage.

### How Is the Project Production and Management of Gifted Students in the Upper and Lower Groups According to Their Program Evaluations?/How Do They Differ?/Is There a Difference Between Them?

The project production and management findings of the upper and lower group participants are summarized in Table 5. Data obtained from “Project report form” confirmed and validated with the other two qualitative data sources used in Table 5.

**Table 5:** Project production and management findings of upper and lower group participants.

	**Project production and management**							**Project/product**
**Group**	***P***	**PE**	**Determining the project topic**	**Specifying the project problem situation**	**Working on the project**	**Finalizing the project**	**Disseminating the project**	
Upper group	1	260	- Doing literature research - Following national and international competitions	- Appropriate for your talent - Preparing a research question	- Compatible with scientific research methods - Doing planning - Utilizing dynamic software	- Carrying out the project according to the plan - Achieving original mathematical generalizations	- Participating in project-based competitions - Making recommendations regarding the project results - Sharing the project results	- Mathematical generalization
	2	241	- Researching the proposals of completed projects	- Solving problem situations that may be encountered in daily life	- Collecting data on the project topic - Determining the programs to be used	- Analyzing the data results - Converting the project results into an original product	- Converting the project results into products - Making recommendations regarding the project results	- Mathematical modeling
	3	241	Attending the cryptology camp	- Appropriate for acquired knowledge - Appropriate for education received	- Planning the budget of the project	- Developing a unique encryption algorithm - Confirming the results	- Applying for a patent related to the original product - Making recommendations regarding the project results	- Cryptology
	4	240	- Orientation to the implementation areas of mathematics	- Appropriate for special talent	- Collecting data - Utilizing dynamic software	- Developing algorithms for the number of moves - Compliance with the work-time schedule	- Making recommendations regarding the project results - Making presentations at congresses	- Algorithms and Logical Design
	5	239	- Making interdisciplinary connections	- Appropriate for mathematical knowledge - Advocating the importance of the project	- Efficient use of facilities	- Compliance with the work-time schedule - Making a geometric proof - Confirming the results	- Participating in project-based competitions - Making recommendations regarding the project results	- Algorithms and logical design
Lower group	1	141	- Following national and international competitions	- Developing an existing project	- Testing hypotheses	- Reporting the results - Using the results in a different situation	- Converting the project results into products - Making recommendations regarding the project results	- Generalizing the results
	2	140	- Obtaining advice from your advisor	- Advocating the importance of the project - Developing the existing one	- Preparing a research question - Utilizing dynamic software	- Reporting the results	- Making a peer presentation - Preparing a poster	- Establishing mathematical relationships
	3	135	- Following different projects	- Preparing a research question	- Collecting data - Utilizing dynamic software	- Reporting the results	- Making a presentation within the scope of the center - Preparing a poster	- Generalizing the results
	4	134	- Obtaining advice from a domain expert - Consulting resource persons	- Consulting resource persons	- Collecting data	- Expanding the scope of the model	- Making a presentation within the scope of the center - Preparing a poster	- A new usage area for an existing model - Project reports
	5	133	- Reviewing examples of projects	- Applying the developed model to different areas	- Gathering data on the project topic	- Expanding the application area of the model	- Making a presentation to peers within the scope of the center	- Extending the application area of the existing model - Posters

When Table 5 is examined, the first column shows the participant group, the other columns show the participants' number (P), the participant's program evaluation score (PE), the project production and management process and categories, respectively, and the last column shows information about the final project/product that emerged. It was concluded that participant number 1 from the upper group on the third line determined the project topic by doing literature research and following international competitions. Project report statements related to this finding were “*As a result of the literature review, questions were encountered about how many different ways the pieces can be placed on the chessboard according to a certain rule and what is the largest square space in these positions”* and “*Within the scope of the project, it was inspired by a problem posed in the internationally held olympiad named the International Mathematical Olympiad (IMO)….”* It was concluded that the same participant determined the problem situation in line with his/her talent and knowledge. The statement related to this finding is “*Based on the problem, new problems were created according to the rules for moving the pieces and the size of the board on which they are placed. …the new problems that were created and the generalization of the solutions given to these problems were worked on.”* It was determined that the participant worked on the project by doing planning and in accordance with the scientific research in the project field. The statement related to this is “*The largest square space formed in the placement of the chess pieces and newly identified types of pieces on the chessboard according to the rules for moving them was examined. It was noticed that these spaces and the number of dimensions are in a certain order. This perceived order was studied. It was investigated whether this order is valid in different dimensions and for different pieces that were identified.”* It was determined that the participant carried out and finalized the project he/she planned by achieving mathematical generalization. The related statement is “*In the solution of the problems, equations were obtained that ensured that the appropriate k values were found for each n. This condition was not provided for k* + *1; on the contrary, it was proven by way of exemplification.”* It was determined that the participant disseminated his/her project by participating in project-based competitions, and the finding related to this was obtained from the statement “*Salih Zeki Mathematics Research Projects Competition”* in the section “Competitions (if any) that the project was entered in and the placings it received.” It was determined that the participant made an original and scientific contribution to the field he/she was doing a project in by making mathematical generalizations as a project/product, and the statement regarding one of the generalizations related to this is “*The existence of* *k* × *k* *squares without a rook in them and the existence of at least one rook in any* (*k* + 1) × (*k* + 1) *square was proven. Therefore, it was concluded that the answer is* $k\;=\;\left\lfloor \sqrt{}\left(n\;-\;1\right)\right\rfloor$*.”*

When Table 5 is examined, was concluded that participant number 5 from the lower group on the last line determined the project topic by reviewing examples of projects. Project report statements regarding this finding are “*It was inspired by a project in which a model was created using the geometric properties of the equilateral triangle and the circle for the design of the system”* and “*Different usage areas for the developed model were considered.”* It was concluded that the same participant determined the problem situation for the application of the developed model to different areas. The statement regarding this finding is “*The circularity of the geometric surface and how the developed model can be utilized in energy production can be determined by surface calculations related to it.”* It was determined that the participant worked on the project by gathering data. The related statement is “*Different results were transferred to the table by calculating the areas of the circle segments and equilateral triangles with the aid of the expressions* *M* = *A* + *B* = π.*x*2.1/6 *and* $N=B=\;\left(x2.\sqrt{}3\right)/$*, respectively. The obtained data were compared.”* The result statement stating that the participant expanded the application area of an already existing model is “*The optimum value was calculated according to the variables. It was demonstrated that the model can also be used in energy production.”* It was determined that the participant disseminated his/her project all around the center, and the relevant finding was obtained from the expression “*The poster prepared was presented at the science fair”* in the section “Whether the project was presented or not.” It was determined that the participant expanded the application area of the existing model as a project/product, and the statement regarding this is “*The scope of the model was expanded in terms of area of use.”*

When the project production and management processes of the participants in the upper and lower groups were compared according to the categories specified in the first line of Table 5, differences were determined. It was determined that in line with their talents, knowledge in their field of expertise, and the literature in their field of expertise, participant number 1 in the upper group specified more innovative project topics than participant number 5 in the lower group. It can be said that both groups were able to determine the project topics, since in the lower group, they were mostly based on projects that had been done before, but that there was a difference in favor of the upper group in terms of depth, originality and quality.

When the problem situation specifications of the upper group participants summarized in Table 5 are examined, it can be stated that compared to the lower group, they were more conscious in terms of orienting toward research questions that they could find answers to in line with their talents. Since the upper group specified problem situations that could be solved in daily life in accordance with their mathematical knowledge and special talents, they produced better quality work in the finalization and dissemination of the project and needed much less advisory support compared to the lower group. It was determined that the upper group successfully reflected their strengths and talents in the project production process compared to the lower group. It can be said by looking at the relevant section of Table 4 and the products produced that while working on the project, the upper group was able to proceed in a much more planned and programmed way, and underwent the process more productively, in comparison with the lower group. The biggest difference between the participants in the upper and lower groups is striking in terms of project results and the products produced. Both groups of participants achieved results, but it is seen that there was a positive difference in favor of the upper group in terms of taking care to confirm or prove the results they achieved. The upper group findings related to mathematical generalization, modeling, cryptology, algorithms and logical design are the findings supporting the fact that the products differed in terms of originality and quality. There are similar differences in the dissemination of the projects in terms of being in favor of the upper group. For example, the fact that dissemination by participant number 5 in the lower group remained within the scope of the center, while the project of participant number 1 in the upper group made it to the nationally prestigious competition finals is a finding that supports this difference. Findings such as the fact that participants from the upper group applied for patents and attended scientific congresses also support this difference.

## Discussion and Conclusion

From the analysis of the gifted students' program evaluation scores, it was concluded that they had positive views about the effectiveness of the implemented program. With this result, the research results related to the gifted students' program satisfaction were confirmed in a sense (Kayişdag and Melekoglu, 2019). In his study, Sak (2011) also concluded that gifted students studying at SACs had high levels of satisfaction with the program they were connected with. Although it was concluded in the quantitative stage of the research that the gifted students had positive views about the effectiveness of the program, it was determined that there was a significant difference between the lower and upper groups in terms of the views that the gifted students expressed. In the study, the aim was to examine what kind of differences were caused between gifted students by the findings obtained in terms of project development, by considering that the quality of the implemented programs directly improved the students' skills in project production and management (Manuel and Freiman, 2017; Sengil-Akar and Yetkin-Ozdemir, 2020). In the qualitative stage of the study conducted in this direction, it was concluded that there were differences in the project production and management processes in favor of the upper group in the categories of determining the project topic, specifying the problem situation, working on the project, finalizing the project, disseminating the project, and the final product, and these results are listed in Table 6.

**Table 6:** Project production and management results in favor of the upper group of gifted students.

**Category**	**Results in favor of upper group**
Determining the project topic	1. Determining more innovative project topics compared to the lower group in line with their talents, their knowledge in their field of expertise, and the literature in their field of expertise. 2. With regard to determining the project topic, differing from the lower group, which mostly went through projects conducted previously, in terms of depth, originality and quality.
Specifying the problem situation	3. Acting more consciously compared to the lower group in terms of orienting toward research questions that they could find answers to in line with their abilities. 4. Since problem situations that could generate solutions in daily life were determined in accordance with their mathematical knowledge and special talents, doing better quality work and with less need for advisory support in the finalization and dissemination processes of the project compared to the lower group.
Working on the project	5. Successfully reflecting their strengths and talents in the project production process compared to the lower group. 6. While working on the project, progressing in a much more planned and programmed way, and undergoing the process more productively compared to the lower group.
Finalizing the project	7. Positive differentiation in the upper group in terms of taking care to verify or prove the results they have obtained.
Disseminating the project	8. Disseminating the project by participating in better attended and more prestigious competition finals, applying for patents, and participating in scientific congresses compared to the lower group.
Project/product	9. Differentiation in terms of originality and quality of products such as mathematical generalization, modeling, cryptology, algorithms and logical design in comparison with the lower group.

When the results presented in Table 6 are examined, it has been determined that all the upper and lower group participants were able to produce projects, but that in the categories specified in their projects, there were differences in favor of the upper group in terms of depth, originality, taking their talents into account, acting consciously, acting in accordance with the plan, participating in more prestigious competitions, and quality.

In the literature, the findings obtained from studies conducted on the effectiveness of programs for the gifted support the conclusion that the project production and management of gifted individuals is associated with the quality of the programs implemented (Manuel and Freiman, 2017; Yu and Yun, 2017; Thahir et al., 2019). As a matter of fact, the programs developed aim to improve and deepen the talents of gifted individuals, and the fact that project production and management is the basis of the principles and guidelines of the gifted education program (MoNe, 2018, 2019) is the reason for the results related to the findings for the project production and management of the gifted students in the upper and lower groups.

The main objectives determined in the gifted education program model are that the program fosters inquisitive and creative thinking, the content of the program is related to daily life, it contributes to the course success of students at school, the lessons are interesting and supported by different methods, how to be a good person is taught, high-level topics are taught, teachers are well-qualified, and the program contributes to the student in different ways (Sak, 2011). When the above-mentioned items are examined in terms of project production and management, it can be said that the great majority of them are among the objectives expected to be achieved by students in the process of developing and conducting a project. By benefiting from project-based programs for the development of their abilities, individuals are given the opportunity to access information, to apply their knowledge, to develop their deep learning, research and scientific thinking skills, to connect with everyday life, to generate different hypotheses, to develop their creativity, to achieve products, and to perform self-evaluation and self-regulation (Renzulli, 2002; Özçelik, 2018).

Today, due to the fact that the individuals who shape the societies are among the gifted students, developed countries attach great importance to the education of gifted students and the development of programs for gifted students so that these students can develop their talents and use their capacities efficiently (Ayvaci and Bebek, 2019). When the gifted education programs implemented in different countries are examined, it can be said that the goals planned to be gained by the gifted students support the students' project development and management skills (MoNe, 2013). Comparing with other studies that are carrying out it in other countries, it has been examined that project producing process of gifted students in terms of their skill, ability, and competence development (Yu and Yun, 2017) and found that those can be developed through project-based programs (Hendriana et al., 2019).

For example; the objectives of the “Gifted Education Program, GEP” implemented in Singapore, which is located in the upper row in international tests (OECD, 2018), are as follows (Yilmaz Bodur and Er, 2019): In-depth learning, interdisciplinary connections, researching real-life problems, higher-order thinking, problem solving, research skills, presentation skill, authentic learning, etc. Although there are different practices for the education of gifted students in each state in Germany, it is stated that the education of gifted students is given importance in general (Fischer and Müller, 2014). It is stated that this importance given is shown by providing academic support from universities in the education of gifted students, giving importance to art courses as well as language education, physics, and mathematics courses, and supporting students to prepare projects and participate in national competitions (MoNe, 2013). Considering the different gifted programs (Neumeister and Burney, 2021), the production of original ideas, products and projects is considered a common basis similar to PPM in the research. The results of this research that was given the opportunity to do an implemented program to the gifted students which are aimed for developing the projects can be considered by other gifted centers. In addition, this research differs from the other researches (Hertzog, 2003; Cho and Lee, 2006; Gavin et al., 2009; Al–zoubi and Rahman, 2015; Redding and Grissom, 2021) in terms of evaluating the impact of a program from the students' projects productions.

Based on the similarity between the main objectives of the gifted education program and the student outcomes expected to be gained in the project production and management process, it can be said that the students who find the gifted program to be effective and of good quality can achieve the specified program objectives more than the students who do not find it to be of good quality, and that consequently, they are more effective and competent in project production and management. It has been determined that students who deal with independent and small group projects, which are at the center of the gifted education program, perceive their projects as interesting and useful and believe that they contribute to their continuing interests and perceptions of pleasure in the future (Reis et al., 2021). In addition, students are the most basic products of the program they evaluate. It can be said that it is possible for gifted students, who are expected to be competent in terms of self-evaluation, to decide on the effectiveness of the program by interpreting the product produced as a result of the program according to the changes that occur in themselves. Project developers' ability to self-organize and achieve goals is greatly enhanced as they benefit from exposure to hard work and environmental support, including trusted relationships with project mentors, teachers, and like-minded peers (Brigandi et al., 2018).

## Recommendations

For future researches, it would be suggesting to study if gifted students' demographic information influence the answers to the Gifted Education Program Evaluations Form and the differences that may exit in the points of view that are given in it. Because; in the quantitative dimension, especially their ages, grade levels, and years of experience at SACs may influence the students' perception of the program and their opinion of it. In the qualitative dimension, in addition to observing the comparisons with the lower group, it is recommended to interpret the project production and management results according to the demographic characteristics of the gifted students. Moreover, it can be recommended that more studies should be conducted on the evaluation of the existing program based on the opinions of gifted individuals. Project-based education programs aim to develop students' high-level thinking and research skills through the projects related to real-life problems that they produce. Therefore, studies should be carried out for the effective use of programs that focus on project production and management aimed at developing the skills of gifted individuals in formal institutions as well as in SACs. Gifted individuals are educated in mixed classes in Turkey. In order for the teaching-learning process in mixed classes to be effective, it can be recommended that teachers should be directed to in-service training related to project production. It can be recommended that pre-service teachers studying at education faculties should be enabled to develop projects or directed to project development courses so that they can support the gifted students they will encounter when they begin their profession. It can be recommended that the programs implemented for gifted students should be designed for normal students as well.

The participants are limited to the gifted students who attended the PPM and STD programs at SACs in Turkey and who are at secondary and high school level as a requirement of these programs. Also, since the participants' project production fields are mathematics, it is recommended that the limitation of the research in terms of field should be taken into account in further researches on project production.

## Data Availability Statement

The raw data supporting the conclusions of this article will be made available by the authors, without undue reservation.

## Ethics Statement

The studies involving human participants were reviewed and approved by Akdeniz University Rectorate Social and Human Sciences Scientific Research and Publication Ethics Committee Decision. Written informed consent from the participants' legal guardian/next of kin was not required to participate in this study in accordance with the national legislation and the institutional requirements.

## Author Contributions

MD and GÖ contributed to conception and design of the study and wrote sections of the manuscript. GÖ organized the database and performed the qualitative analysis. MD performed the statistical analysis. Both authors contributed to manuscript revision, read, and approved the submitted version.

## Conflict of Interest

The authors declare that the research was conducted in the absence of any commercial or financial relationships that could be construed as a potential conflict of interest.

## Publisher's Note

All claims expressed in this article are solely those of the authors and do not necessarily represent those of their affiliated organizations, or those of the publisher, the editors and the reviewers. Any product that may be evaluated in this article, or claim that may be made by its manufacturer, is not guaranteed or endorsed by the publisher.
